# Environmental Exposure and Leptospirosis, Peru

**DOI:** 10.3201/eid1006.030660

**Published:** 2004-06

**Authors:** Michael A.S. Johnson, Hannah Smith, Priya Joseph, Robert H. Gilman, Christian T. Bautista, Kalina J. Campos, Michelle Cespedes, Peter Klatsky, Carlos Vidal, Hilja Terry, Maritza M. Calderon, Carlos Coral, Lilia Cabrera, Paminder S. Parmar, Joseph M. Vinetz

**Affiliations:** * Asociación Benéfica Prisma, Lima, Peru;; † Johns Hopkins Bloomberg School of Public Health, Baltimore, Maryland, USA;; ‡ U.S. Naval Medical Center Research Detachment, Lima, Peru;; § Universidad Peruana Cayetano Heredia, Lima, Peru;; ¶ Ministry of Health, Iquitos, Peru;; # University of California San Diego School of Medicine, La Jolla, California, USA

**Keywords:** Leptospirosis, Peru, Tropical Medicine, Rural Population, Epidemiology, Serology, Environmental Health

## Abstract

Human infection by leptospires has highly variable clinical manifestations, which range from subclinical infection to fulminant disease. We conducted a population-based, cross-sectional seroepidemiologic study in Peru to determine potential relationships of environmental context to human exposure to *Leptospira* and disease associated with seroconversion. Three areas were studied: a flooded, urban slum in the Peruvian Amazon city of Iquitos; rural, peri-Iquitos villages; and a desert shantytown near Lima. Seroprevalence in Belen was 28% (182/650); in rural areas, 17% (52/316); and in a desert shantytown, 0.7% (1/150). *Leptospira*-infected peridomestic rats were found in all locales. In Belen, 20 (12.4%) of 161 patients seroconverted between dry and wet seasons (an incidence rate of 288/1,000). Seroconversion was associated with history of febrile illness; severe leptospirosis was not seen. Human exposure to *Leptospira* in the Iquitos region is high, likely related both to the ubiquity of leptospires in the environment and human behavior conducive to transmission from infected zoonotic sources.

Leptospirosis is a zoonotic disease of global importance (1–5) that occurs in both urban and rural settings (2,6–8) and causes both endemic and epidemic illness, including pulmonary hemorrhage and death (7,9–11). Transmission of *Leptospira* and the clinical expression of leptospirosis seem to vary in different environmental and socioeconomic contexts. Epidemic leptospirosis associated with pulmonary hemorrhage, renal failure, and jaundice seems to predominate in the urban setting, where baseline clinical immunity in humans is likely to vary (7,8,12–15). In contrast, a substantial prevalence of seropositivity associated with subclinical leptospiral infection has been shown in many rural places throughout the developing world, including, for example, Nicaragua (16).

The environment of Iquitos, Peru, in the Amazon Basin, is ideal for the transmission of *Leptospira* with its hot, humid tropical conditions and dense human and potential mammalian reservoir populations (17). We have observed that ≈30% of patients in this region seen with acute undifferentiated fever have serologic results suggestive of acute leptospirosis (microscopic agglutination test with titers >1/400, seroconversion, or fourfold rise in titer; Vinetz et al. unpub. data).

Our objective was to determine potential relationships of environmental context to human exposure to *Leptospira*. A cross-sectional, population-based seroepidemiologic study was conducted in three contrasting epidemiologic contexts in Peru, where leptospirosis transmission would be predicted to be high (Belen, an urban slum in Iquitos), intermediate (rural peri-Iquitos villages), and low (the Pampas de San Juan de Miraflores, a desert shantytown outside of Lima). Peridomestic rats, one potentially important source of leptospiral transmission, were also surveyed for the leptospiral carrier state to assess their potential for transmission.

## Patients and Methods

### Study Sites

**Figure 1:**
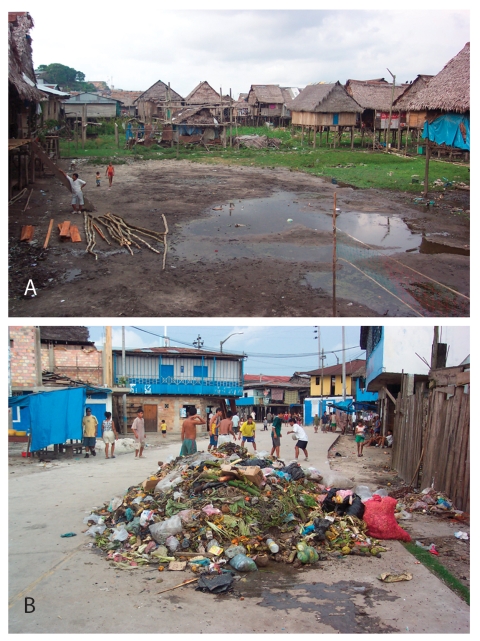
Typical views of the residential areas of Belen, Iquitos, Peru. A. Houses near the river edge are built on stilts. B. Conditions abound for the proliferation of peridomestic rats in the same places where people live and play. This view is adjacent to the major market area of Belen, where *Rattus* spp. are commonly observed, even during the day in the middle of commercial activities.

Iquitos, Loreto, is located in the Amazon rainforest of northeastern Peru. Its environment is tropical: rainfall averages 288 cm/year; temperatures range from 21.8°C to 31.6°C. The region has a population of ≈400,000. Belen (Figure 1), an urban slum area of Iquitos on the floodplain of the Itaya and Amazon Rivers, annually floods during January to May from Andean run-off. Many residences are built on floats; during flooding, these houses rise with the river. Belen has a population of 40,000, primarily mestizos of mixed European and Amerindian ancestry (Dirección Regional de Salud, Iquitos, Peru, pers. comm.). Main occupations include fishing, small-scale commerce, and informal day labor.

**Figure 2:**
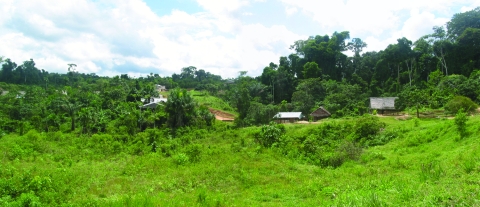
A typical view of a rural village area near Iquitos. Near Villa Buen Pastor, located 21 km along the major (unfinished) road that leads from Iquitos to Nauta, substantial secondary growth of forest is evident after removal of primary forest for human agricultural and living activities. One must walk approximately 1–2 km from the road to get to the village and a further 1–2 km from Villa Buen Pastor to Moralillo, another village studied in this report.

The peri-Iquitos rural communities studied were Moralillo, Villa Buen Pastor, and San Carlos, located 16, 21, and 34 km, respectively, south of the center of Iquitos (Figure 2). Combined, the population of these villages at the time of sampling was 1,197, mainly mestizo. These villages are rural, located upriver away from the city. Most inhabitants live in poor housing with electricity but without household connections to water or sewer systems. The primary occupation is small-scale agriculture. These villages do not flood seasonally because they are above the floodplain. Water is obtained from village pumps or springs. Residents typically bathe in local rivers or ponds.

Las Pampas de San Juan de Miraflores is a pueblo joven (shantytown) located in the desert 25 km south of Lima. The population at the time of sampling was 40,000. Basic demographic and environmental features of this area have been previously described (18–20). Residents are primarily mestizo immigrants from the Peruvian Andes who earn money through informal day labor. Annual rainfall averages <1 cm.

## Study Design

### Belen

A census of 3,704 people from seven communities was conducted and coded in February 2001. The census was divided into four age groups before random selection: 1) 1–5 years; 2) 6–11 years; 3) 12–17 years; and 4) 18–65 years. From the last group, 1,210 adults were randomly selected to participate in a seroprevalence survey from February to October 2001. Eighty children from each of groups 1 to 3 were also recruited. Standardized questionnaires were completed, including questions pertaining to potential household, occupational, and social exposures. Serum was collected and preserved at –20°C for serologic analysis. A subgroup of participants tested in February and March 2001 were retested from July to October 2001 as an incidence cohort.

Rats were collected from a random selection of houses within the same communities as well as other areas of Belen during November 2001 through May 2002 by using Tomahawk (Tomahawk Live Trap Co., Tomahawk, WI) and back-break traps. Kidneys were preserved at –20°C in 0.1 mol/L Tris-HCl, pH 8.0 for subsequent testing.

### Rural Communities

Data on residents of Villa Buen Pastor, Moralillo, and San Carlos were coded; a census was conducted, and samples were obtained from study participants from February 2000 to August 2000. A total of 650 serum samples were collected from 316 randomly selected participants <65 years of age. Participants were afebrile and apparently healthy.

### San Juan de Miraflores

A population of 38,721 was coded and a census conducted in April 1997. During the same period, serum samples were collected from 150 randomly selected participants <65 years of age. Participants were afebrile and apparently healthy. Rats from a random selection of houses were trapped live with Tomahawk traps.

### Laboratory Assays

A combined immunoglobulin (Ig) M + IgG assay (INDX IVD Leptospira Microwell ELISA, PanBio INDx, Inc., Baltimore, MD) was used to obtain evidence of leptospiral exposure from serum samples. The assay uses *Leptospira biflexa* serovar patoc I as antigen to detect genus-specific antibodies. Leptospiral exposure was defined as positivity on an enzyme-linked immunosorbent assay (ELISA), according to the manufacturer’s instructions (optical density at 1/100 dilution of sample greater than or equal to weak positive control). ELISAs were performed twice with identical results.

DNA was extracted from rat kidneys by using a published procedure based on a phenolchloroform-isoamyl alcohol-extraction method (21). The presence of *Leptospira* DNA was assessed by a polymerase chain reaction-based assay (PCR) using G1/G2 primers (22).

The chi-square test was used, or the Fisher exact test, when mandated by sparse data, to compare groups for categorical outcomes. For nonparametric data, either the Student *t* test or the Mann-Whitney U test was used. Associations between seropositivity to *Leptospira* spp. and study variables were analyzed by both univariate and stratified analysis by using odds ratios (ORs). Because the study population included sets of persons for each household, data were presumed to violate the standard logistic regression assumption of independent response probabilities across observations. To avoid potential underestimation of standard errors (23), we estimated ORs by using the logistic-binomial random effects model for distinguishable data from the EGRET software package (Cytel Software Corporation, Cambridge, MA). This model includes a random effects parameter, based on the variability of average outcome probabilities across households, which measure a residual household effect on the probability of having leptospirosis. ORs were adjusted for the effect of household. Confidence intervals (CI) are 95%; p values <0.05 were considered significant and two-sided. Other statistical analysis was performed with Stata v. 7.0 (Stat Corp., College Station, TX).

This study was approved by the Dirección de Salud, Iquitos, Peru; the Ethical Committee of Asociación Benéfica Prisma, Lima, Peru; and the Johns Hopkins Bloomberg School of Public Health Committee on Human Research, Baltimore, Maryland. Written informed consent was obtained from each participant. Guidelines for human experimentation according to institutional, U.S. federal, and Peruvian standards were followed.

## Results

### Demographic Description of Study Populations

In Belen, 650 people from 386 households participated in the study (Table 1); this figure represented 18% of the population and 60% of the households. The median number of persons per household was 5.8 (range 1–21). In the rural communities, 316 participants (26% of local population) were enrolled. The median number of persons per household was 6.4 (range 1–24). In Las Pampas de San Juan de Miraflores, 150 (0.4% of the local population) people were sampled. The median number of people per household in Las Pampas was 5.4 (range 1–14).

**Table 1:** Site description and demographic characteristics of the study population

Feature	Pampas San Juan	Rural communities^a^	Belen
Study period	April 1997	Feb–Jun 2000	Feb–Oct 2001
Population	38,721	1,197	3,704
No. households selected (%)	7,744 (20)	132 (56)	386 (60)
No. participants tested (%)	150 (0.4)	316 (26)	650 (18)
Sample population			
Female (%)	57 (38)	152 (48)	356 (55)
Age >18 y (%)	64 (43)	170 (54)	533 (82)
Mean (SD) age, y	17.5 (14.5)	24.9 (17.9)	30 (15.2)
Median	11	22	29
Mean (SD) no. persons per household	5.7 (2.2)	2.4 (1.6)	1.7 (0.9)
Range	3–14	1–9	1–5

^a^Combined populations of the villages of San Carlos, Moralillo, and Buen Pastor in the peri-Iquitos area.

### Prevalence of Leptospiral Seropositivity

**Figure 3:**
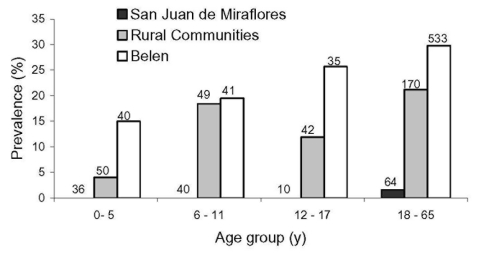
Prevalence of antileptospiral immunoglobulin (Ig) M/IgG antibodies by age group. Number above each bar is the sample size for the specified age group and site. The trend of increasing prevalence by age is significant for Belen and the rural communities (p = 0.018 and p = 0.012, respectively).

In Belen, 182 (28.0%) of 650 people were positive for anti-*Leptospira* antibodies (Table 2). No sex differences in seropositivity rates were evident. Age groups in Belen did not differ statistically (children versus adults; p = 0.122), but a significant trend for seropositivity with age was evident (Figure 3, p = 0.018).

**Table 2:** Leptospirosis seroprevalence among residents by study site

	Pampas San Juan % (pos/total)^a^	Rural communities^b^ % (pos/total)	Belen % (pos/total)	Comparison among all sites	Comparison of Belen vs. rural community
Overall^c^	0.7 (1/150)	16.5 (52/316)	28.0 (182/650)	p <0.001	p <0.001
Sex					
F	0.0 (0/57)	15.8 (24/152)	29.6 (105/356)	p <0.001	p = 0.001
M	1.1 (1/93)	17.1 (28/164)	26.4 (77/292)	p <0.001	p = 0.024
M vs. F comparison	p = 0.999	p = 0.758	p = 0.428		

^a^Leptospirosis prevalence (number of positive cases/total of persons tested). ^b^San Carlos, Moralillo, and Buen Pastor. ^c^Optical density >0.500.

In the rural peri-Iquitos communities, the rate of seropositivity was lower than in Belen (16.5% vs. 28.0%, p < 0.001, Table 2). No sex difference in seropositivity (17.1% vs. 15.8%; p = 0.758) was evident. Although 6- to 11-year-old children had a higher prevalence than 12- to 17-year-old children, overall seroprevalence increased with age, from 4.0% in those <5 years of age to 21.2% in persons 18–65 years (Figure 3, p = 0.012). In Las Pampas de San Juan de Miraflores, 1 of 150 participants was seropositive (Table 2), a finding that yielded an estimated prevalence of 0.7%.

### Incidence of Leptospiral Seroconversion

In Belen, 195 participants (ages 18–65 years) tested in the first 2 months of the prevalence study were retested ≈6 months later; 161 were initially negative. On retesting, 20 (12.4%; 11 women, 9 men) seroconverted from negative to positive (IgM + IgG). Median time between sampling was 157 days, the approximate interval between flooding seasons. The estimated incidence rate of seroconversion was 288/1,000 people per year. Of 34 persons who had a positive first sample and were retested, 18 (53%) seroreverted to negative, a finding that demonstrates that IgM + IgG ELISA–detectable antibodies are short-lived, consistent with results of previous studies (4,24–26).

Univariate analysis showed that history of fever in the previous 6 months was associated with seroconversion (OR = 3.29, 95% CI 1.19 to 9.12, p = 0.022), a finding that remained significant after controlling for age and sex (OR = 3.32, 95% CI = 1.18 to 9.29, p = 0.023). Severe leptospirosis did not develop in any patient.

### Risk Factors for Leptospiral Seropositivity

Univariate analysis demonstrated several potentially modifiable risk factors (Table 3), including river bathing, owning a television, living close to the river, and living in San José, one area of Belen where sanitation is particularly poor. Particular animals present in the home or workplace were not found to be significant risk factors. Educational level was associated with protection when a trend analysis was used (p = 0.032). By multivariate analysis, risk factors were not wearing shoes in the field and living in San José.

**Table 3:** Risk factors associated with leptospiral seropositivity, Belen^a^

Risk factor	Prevalence		Incidence (n = 158)	
	OR (95% CI)	p	RR (95% CI)	p
Living in San José	1.90 (1.27 to 2.83)	0.002	1.88 (0.83 to 4.23)	0.191
Bathing in the river	1.75 (1.09 to 2.82)	0.021	2.70 (0.83 to 8.78)	0.132
Living near the river	1.58 (1.07 to 2.32)	0.022	1.94 (0.84 to 4.47)	0.149
Owning a television	1.55 (1.01 to 2.38)	0.043	1.56 (0.66 to 3.69)	0.438
Fisherman (male)	1.57 (0.86 to 2.87)	0.141	1.94 (0.73 to 5.18)	0.250
Not wearing shoes in the field	1.63 (0.85 to 3.13)	0.139	2.29 (0.93 to 5.59)	0.135
Drinking primarily river water	1.28 (0.82 to 2.01)	0.272	0.91 (0.35 to 2.40)	0.999
Rats in the workplace	1.14 (0.52 to 2.48)	0.740	0.79 (0.12 to 5.18)	0.999
Rats in the home	1.36 (0.00 to 3.61)	0.538	NA	NA
Dogs in the workplace	1.12 (0.66 to 1.90)	0.684	1.72 (0.69 to 4.31)	0.317
Dogs in the home	0.67 (0.43 to 1.02)	0.063	1.06 (0.46 to 2.44)	0.891
Pigs in the home	0.77 (0.38 to 1.53)	0.452	0.44 (0.06 to 3.06)	0.699
Education (minimal vs. secondary)	1.37 (0.90 to 2.05)	0.139	1.59 (0.56 to 4.51)	0.368
Education (secondary vs. superior)	2.63 (0.58 to 11.96)	0.207	NA	NA
Education (minimal vs. superior)	3.60 (0.81 to 15.99)	0.092	NA	NA
Positive household member	1.18 (0.75 to 1.85)	0.468		
Multivariate logistic regression for independent risk factors				
Living in San José	2.17 (1.39 to 3.37)	0.001		
Not wearing shoes in the field	2.17 (1.1 to 4.05)	0.015		

^a^OR, odds ratios; RR, relative risk; CI, confidence interval. CIs and ORs were calculated for adults only (>18 years of age). NA, not applicable.

### Prevalence of Leptospiral Infection in Rats

Of 234 rats (151 [64.0%] *Rattus rattus*, 83 [36%] *R. norvegicus*) trapped in Belen, 25 (10.6%) were positive for *Leptospira* by PCR (Table 4). *R. norvegicus* were more frequently infected than *R. rattus* (21.7% vs. 4.6%, p <0.001). While PCR positivity increased with rat age (infantile rats 5.9%, juveniles 8.8%, adults 12.0%), this apparent trend was not significant (p = 0.328). Of rats in San Juan de Miraflores (all *R. norvegicus*), 2 of 41 (4.9%) were PCR-positive for *Leptospira* (1/25 males, 1/16 females). Both infections were confirmed by culture. Isolates were not further identified.

**Table 4:** Characteristics of rats in Belen and Las Pampas de San Juan de Miraflores^a^

Risk factor	Pampas San Juan % (pos/total)^b^	Belen % (PCR positive/total)^b^	Comparison of sites p value
No. rats tested	4.9 (2/41)	10.6 (25/235)	p = 0.392
Sex^c^			
F	6.3 (1/16)	11.6 (15/129)	p = 0.999
M	4.0 (1/25)	8.5 (8/94)	p = 0.683
M vs. F comparison	p = 0.999	p = 0.594	
Age groups			
Adult	3.9 (2/41)	12.0 (18/150)	p = 0.255
Young (prepubescent)	0	8.8 (6/68)	p = NA
Infant	0	5.9 (1/17)	p = NA
Trend	p = NA	p = 0.328	
Species			
*Rattus norvegicus*	4.9 (2/41)	21.7 (18/83)	p = 0.019
*R. rattus*	0	4.6 (7/151)	p = NA
Comparison of rat species	p = NA	p <0.001	

^a^PCR, polymerase chain reaction; NA, not applicable. ^b^Leptospirosis prevalence (number of positive rats/total of rats tested). ^c^Sex was not known for 12 infants.

## Discussion

This study produced two major findings. First, human exposure to *Leptospira* in sites with contrasting ecologic features depended more on environmental context than the prevalence of infection in peridomestic zoonotic sources. Second, in Belen, a flood-prone urban slum area of Iquitos where exposure to *Leptospira* was common, seroconversion was associated with symptomatic, but not severe, disease.

The evidence for human leptospiral infection in Iquitos is different from what is typically reported from the tropics (9). In areas where *Leptospira* would be predicted to be common, epidemics are usually described in the context of outbreaks caused by events such as the flooding associated with the Nicaraguan epidemic of 1995 (9), not in the leptospirosis-endemic setting such as we describe here. Previously described risk factors elucidated in other tropical contexts were investigated in Belen (Table 3). Most had no pertinence in Belen because of their ubiquity in this tropical, urban slum. Only two factors were significant in multivariate analysis: not wearing shoes when working in the field and living in the San José area of Belen.

Two risk factors for leptospiral seropositivity of particular interest were education and living close to the river, specifically in San José. Prevalence of antileptospiral antibodies decreased with increased education, most likely associated with a greater degree of personal hygiene. Living in San José was a significant positive risk factor in the prevalence study by both univariate and multivariate analysis and was a risk factor in the incidence cohort as well. Rats also had higher rates of infection in San José than elsewhere in Belen, a finding that suggests that, within the overall highly disease-endemic area of Belen, smaller microhabitats may have even higher rates of leptospirosis transmission.

The overall high prevalence and incidence of leptospirosis transmission found in the absence of a disease outbreak, as well as similar rates of seropositivity in both sexes, indicate the high level of risk for infection in this area. In most epidemiologic studies of leptospirosis, infection rates differ for men and women, related to gender-specific activities (16,27–31) and associated leptospiral serovars (32). In the Iquitos study populations, the prevalence and incidence were very similar between the sexes, although the occupations of men and women were very different. Furthermore, the relationship between seropositivity and age shows the same trend as found in studies of other, largely native, inhabitants of *Leptospira*-endemic regions (33,34). These data support the hypothesis that continuous exposure throughout life may result in an age-dependent increase in leptospiral seropositivity.

Limitations of this study must be considered. First, the different components of the study took place during different periods, rather than concurrently. Since the study sites in the Iquitos region are climatologically different from those in Lima and no disease outbreaks were observed in either location, our results likely reflect a reasonable estimate of endemic leptospiral transmission. Second, the high seropositivity rate in the absence of observed disease in Iquitos could relate to potential cross-reactivity of currently used ELISA antigens rather than true differences. However, the reproducible finding of 0.7% seroprevalence in a desert community where very little leptospiral infection might be predicted (San Juan de Miraflores), compared to 28.0% in Belen, suggests that the ELISA we used was specific, consistent with previous studies that used similar testing procedures (9,16,24,35,36). In addition, we found that 53% of persons with positive ELISA results seroreverted to negative within 6 months. This finding is consistent with known short-lived serologic responses to leptospiral lipopolysaccharide antigens (4). Third, several hypotheses might explain the observed differences between study sites. For example, the intrinsic pathogenicity of *Leptospira* in the study region may differ. Whether the immunogenetics of the human immune response to *Leptospira* differs between populations is unknown. The prevalence in rats may also have been underestimated. The G1/G2 primer set used to detect this genus in rat kidneys detects most, but not all, leptospires. Our overall findings are also consistent with previous studies (33,34), in which high prevalence rates of antileptospiral antibodies have been found despite the absence of observed severe disease. Thus, protective immunity against severe disease from repeated infection may develop in persons in areas with ubiquitous leptospirosis transmission.

The high rate of exposure to *Leptospira* in Iquitos, and the likely ubiquity of these organisms in the environment, suggests that implementing control measures to prevent leptospirosis in this setting might be difficult. The seeming absence (or at least very low rates) of severe leptospirosis in this region suggests the possibility that protective immunity may develop in this population. Larger prospective, population-based studies will be necessary to test these possibilities.
